# Self-reported skin severity and quality of life in systemic sclerosis: multicentre validation of PASTUL

**DOI:** 10.1093/rheumatology/keae561

**Published:** 2024-10-14

**Authors:** Julia Spierings, Paco M J Welsing, Seda Colak, Helen Quah, Francesco Del Galdo, Ariane L Herrick, Michael Hughes, John D Pauling, Voon H Ong, Christopher P Denton

**Affiliations:** Centre for Rheumatology and Connective Tissue Diseases, UCL Medical School Royal Free Campus, London, UK; Department of Rheumatology & Clinical Immunology, University Medical Centre Utrecht, Utrecht, The Netherlands; Department of Rheumatology & Clinical Immunology, University Medical Centre Utrecht, Utrecht, The Netherlands; Leeds Institute of Rheumatic and Musculoskeletal Medicine, University of Leeds, Leeds, UK; Centre for Rheumatology and Connective Tissue Diseases, UCL Medical School Royal Free Campus, London, UK; Leeds Institute of Rheumatic and Musculoskeletal Medicine, University of Leeds, Leeds, UK; Division of Musculoskeletal & Dermatological Sciences, The University of Manchester & Northern Care Alliance NHS Foundation Trust, Manchester Academic Health Science Centre, Manchester, UK; NIHR Manchester Biomedical Research Centre, Manchester University NHS Foundation Trust, Manchester Academic Health Science Centre, Manchester, UK; Division of Musculoskeletal & Dermatological Sciences, The University of Manchester & Northern Care Alliance NHS Foundation Trust, Manchester Academic Health Science Centre, Manchester, UK; Department of Rheumatology, North Bristol NHS Trust, Bristol, UK; Centre for Rheumatology and Connective Tissue Diseases, UCL Medical School Royal Free Campus, London, UK; Centre for Rheumatology and Connective Tissue Diseases, UCL Medical School Royal Free Campus, London, UK

**Keywords:** systemic sclerosis, scleroderma, skin, patient reported outcome, quality of life

## Abstract

**Objectives:**

The aim of this study was to validate the Patient self-Assessment of Skin Thickness in Upper Limb (PASTUL) questionnaire in SSc and assess impact of skin involvement on health-related quality of life (HRQoL).

**Methods:**

Participants were included in four UK centres. PASTUL specifies a grading of skin at eight sites corresponding to the modified Rodnan Skin Score (mRSS). Construct validity was assessed by comparing PASTUL scores with mRSS. HRQoL was evaluated with EuroQoL 5 dimension 5 levels (EQ5D5L) and Leeds SSc QoL questionnaires. Additionally, correlation between PASTUL and Scleroderma Skin Patient-Reported Outcome (SSPRO) was explored. Follow-up was 12 months.

**Results:**

In total, 196 participants were included, mean age was 56.4 years (s.d. 13.9), 80.6% female (*n* = 158), mean disease duration 11.9 years (s.d. 9.9), 110 (56.1%) had lcSSc and 81 (41.3%) dcSSc. PASTUL and upper limb mRSS were well correlated at baseline, 6 and 12 months [intraclass correlation coefficients (ICC) = 0.67, 0.78 and 0.62, *P* < 0.001]. Test–retest reliability was good (ICC = 0.83, *P* < 0.001). There was a stronger correlation between PASTUL and upper limb mRSS in dcSSc compared with lcSSc (0.69 *vs* 0.51, *P* < 0.001). In participants with early disease (<4 years) PASTUL was moderately correlated with HRQoL (r = 0.53, *P* < 0.001); correlations were weaker in the whole group. Mean time to do the PASTUL self-assessment was 5.0 min (s.d. 3.7).

**Conclusion:**

PASTUL is a feasible outcome tool that adds to assessments such as SSPRO. Skin thickening is correlated with HRQoL, particularly in early disease.

Rheumatology key messages

- PASTUL and upper limb mRSS correlate well cross-sectionally and test–retest reliability is good.
- PASTUL correlates with HRQoL (EuroQoL VAS and Leeds SSc QoL).
- PASTUL can be used as a complementary outcome measure to skin symptoms measured with SSPRO.

## Introduction

SSc is a rare autoimmune CTD [1]. Skin fibrosis is a central feature of SSc. However, not much is known about the impact of skin involvement on quality of life, from a patient’s perspective.

Evaluation of skin involvement is central in clinical practice. Skin thickness is routinely assessed by the modified Rodnan Skin Score (mRSS) [2, 3] and has been found to be associated with internal organ manifestations and mortality [4]. Therefore, skin thickness assessed with the mRSS is often used as an outcome measure in clinical trials [5–7] and is a valuable tool in routine practice.

Remote consultations were widely implemented in response to the COVID-19 pandemic [8, 9], and home-monitoring of disease activity is increasingly explored both for clinical practice and in decentralized clinical trials. Patient-reported outcome (PRO) instruments could play a valuable role in remote monitoring.

In a recent pilot study, we explored ways to physically assess skin in SSc with the Patient self-Assessment of Skin Thickness in Upper Limb (PASTUL) questionnaire [10]. We demonstrated that using the PASTUL questionnaire is a feasible and easy approach for evaluation of skin thickness in SSc patients.

The aim of this study was to further validate the PASTUL questionnaire in a larger population across different scleroderma centres in UK and to specifically investigate the relation between skin involvement and health-related quality of life (HRQoL). This is important because meaningful change in skin severity may be expected to impact on people with SSc and supports the utility of PASTUL in routine practice and as a potential outcome measure that could be tested in future clinical trials that could be both simpler to collect and more directly linked to feeling and function.

## Methods

### Study setting and participants

Adult patients with an established diagnosis of SSc fulfilling the 2013 ACR-EULAR criteria for SSc [11] who were treated at the Royal Free Hospital London, North Bristol NHS Trust, Northern Care Alliance NHS Foundation Trust (Salford), and Leeds Institute of Rheumatic and Musculoskeletal Medicine, UK, were invited to participate. Only participants at the Royal Free Hospital London were followed for 12 months to evaluate sensitivity to change.

Informed consent was obtained from all participants. The study was approved by the London-Fulham Research Ethics Committee. Patients received verbal information about the study and if they agreed to participation they received additional written information, explaining the aim of the development and evaluation of the PASTUL questionnaire and detailed instructions about skin-assessment with relevant images. Participants could complete the surveys online using the electronic data capture platform Castor.

When participants visited the hospital for their appointment, the mRSS was performed by an experienced rheumatologist or trained research fellow (M.H., J.D.P., V.H.O., C.P.D., F.D.G., S.C.) after participants completed the questionnaire themselves and without seeing the self-assessed score.

### The PASTUL questionnaire

The PASTUL questionnaire is a simple grading of skin as normal, mild, moderate or severely thickened at eight sites of upper limb that correspond to mRSS assessment [2, 10, 12] (Supplementary Material S1, available at *Rheumatology* online). To simplify the assessment, we asked participants to grade the maximum score of an anatomical area. Assessed grades were converted to an integer scale (0, 1, 2, 3) per area in order to align the score with the mRSS.

Instructions on self-assessment of skin used in the pilot study were optimized and discussed with 10 SSc patients in focus interviews (Supplementary Materials S2 and S3, available at *Rheumatology* online).

### Data collection and measures

Demographic and clinical information was collected including age, sex, disease subtype and disease duration. The mRSS was done in participants visiting the hospital for routine treatment or follow-up. Both the upper limbs (0–24) and total mRSS (0–51) scores were registered. To align with the patient assessment, the mRSS was done using the maximum score at each anatomical area. Daily functioning was assessed with the validated Scleroderma HAQ Disability Index (HAQ-DI). HAQ-DI scores ranging from 0 (no disability) to 3 (maximal disability) and six visual analogue scale (VAS) scores (from 0–100) for the domains pain, gastrointestinal complaints, breathing, RP, digital ulcers and general limitations [13].

HRQoL was assessed by the Leeds Systemic Sclerosis Quality of Life Questionnaire (Leeds SSc QoL) and EuroQoL 5 dimension 5 levels (EQ5D5L). Leeds SSc QoL is a questionnaire containing of 29 true/not true answers, with an overall score between 0–29 [14, 15]. EQ5D5L consists of five questions with five level answers which can be summarized in an overall ‘utility’ score ranging from 0–1, with 0 representing death and 1 perfect health and a VAS about the overall health today from 0–100, with 0 representing the worst general health one could imagine and 100 the best [16].

Additionally, skin-related quality of life was assessed using the Scleroderma Skin Patient-Reported Outcome (SSPRO) questionnaire [17]. This 18-item PRO measure evaluates skin symptoms in SSc using a 6-point scale (‘not at all’ to ‘very much’) in four different dimensions: physical effects (5 items), physical limitations (4 items), emotional effects (6 items) and social effects (3 items). Correlations were calculated using the total score and all four subscales.

Participants from Leeds Institute of Rheumatic and Musculoskeletal Medicine also completed the Cochin Hand Function Scale (CHFS) [18]. This questionnaire contains 18 questions concerning daily activities, each question being scored from 0 (performed without difficulty) to 5 (impossible to do), with a total score (sum of all questions) ranging from 0 to 90. A total CHFS of 26 and up was regarded as hand function limitation [19].

All data (mRSS, clinical data and PRO assessments) were recorded on the same day that PASTUL was performed.

### Feasibility and test–retest reliability

Feasibility was evaluated in a subgroup of participants by scoring relevance (ranging from extremely irrelevant to extremely relevant), clarity and practical difficulty (ranging from very difficult to very easy) of the PASTUL on a 5-point Likert scale. Participants were also asked to report the time needed to do a self-assessment of their skin (in minutes). This subgroup also repeated the PASTUL 7–14 days after the first assessment to determine the test–retest reliability.

### Sensitivity to change

Sensitivity to change of the PASTUL questionnaire and the impact of change of skin thickness on HRQoL was assessed in the participants at the Royal Free Hospital. Participants completed the abovementioned questionnaires at inclusion and after 6 and 12 months. The mRSS was scored at these timepoints as well. The correlation between a relevant change in mRSS upper limbs and PASTUL (decrease, stable or correlation) was assessed for the 6 months follow-up interval (baseline and 6 months, and 6 months and 12 months) and 12 months follow-up using Cohen’s Kappa. A change of >3 in PASTUL and mRSS upper limbs was used to define a relevant change.

### Data analysis

Data were analysed using SPSS 25 (IBM, USA). Descriptive statistics (mean, s.d. or median, Q1, Q3 for continuous normally or non-normally distributed variables, respectively, and frequency with percentage for categorical variables) were used to describe sex, disease subtype, antibody profile and disease duration (mean, s.d.) for all participants.

Construct validity was evaluated by examining the correlation between the PASTUL and mRSS (upper limbs) using intraclass correlation coefficients (ICC).

Hypotheses were generated *a priori* to examine the extent to which baseline scores (construct validity) of PASTUL were associated with the upper limb mRSS: PASTUL scores have a good correlation with upper limb mRSS.

Test–retest reliability was estimated using intraclass correlation coefficient. To evaluate feasibility, mean values were obtained for relevance, understandability and performance Likert scores, and completion time. Floor and ceiling effects for the PASTUL were defined by adopting the conventional 15% threshold for the highest and lowest scores, respectively.

Correlations between PASTUL and PROs—SSPRO, Leeds SSc HRQoL, EQ5D5L and HAQ-DI—were explored using Pearson’s or Spearman’s correlation coefficient.

Coefficients were interpreted as follows, for the Pearson’s correlation coefficient: 0–0.19 = negligible, 0.2–0.39 = weak, 0.4–0.59 = moderate, 0.6–0.79 = strong, 0.8–1.0 = very strong; and for the ICC: <0.5 = poor, 0.5—0.75 = moderate, 0.75–0.9 = good, >0.90 = excellent [20, 21].

Descriptive statistics were used to describe mean change over time. Sensitivity to change was assessed using the change of PASTUL score and mRSS score [nominal (>3 points change, <3 points change or stable)] between baseline, and 6 and 12 months.

## Results

Across four centres, 236 patients were invited to participate. In total, 196 patients completed the questionnaires and were included for analysis. Mean age was 56.4 years (s.d. 13.9), 80.6% female (*n* = 158), mean disease duration 11.9 years (s.d. 9.9), 110 (56.1%) had lcSSc and 81 (41.3%) dcSSc. The mean EuroQol index was 0.61 (0.27), HAQ-DI 2.13 (s.d. 0.68), mean Leeds SSc QoL 16.2 (s.d. 8.0) and mean SSPRO 45.6 (s.d. 27.9). All characteristics are shown in Table 1.

**Table 1.** Patient characteristics

	Total *N* = 196
Male sex, *n* (%)	38 (19.4)
Age (mean, s.d.)	56.4 (13.9)
Disease subset, *n* (%)	
lcSSc	110 (56.1)
dcSSc	81 (41.3)
Unknown/undetermined, *n* (%)	5 (2.6)
Disease duration, years (mean, s.d.)	11.9 (9.9)
Digital ulcers at inclusion, *n* (%)	74 (37.8)
Auto-antibodies, *n* (%)	
ACA	63 (32.1)
ATA	39 (19.9)
ARA	19 (9.7)
Other	47 (24.0)
ANA only	12 (4.6)
No antibodies	9 (4.6)
Unknown	7 (3.6)
Baseline mRSS (mean, s.d.)	8 (8)
EQ5D5L index (mean, s.d.)	0.61 (0.27)
EQ5D VAS (mean, s.d.)	56.8 (21.9)
Leeds SSc QoL (mean, s.d.)	16.2 (8.0)
SSPRO total score (mean, s.d.)	45.6 (27.9)
SSPRO subdomains (mean, s.d.)	
Physical effects	13.7 (6.7)
Physical limitations	9.8 (8.0)
Emotional effects	16.7 (10.5)
Social effects	5.3 (5.5)
HAQ-DI (mean, s.d.)	2.13 (0.68)
HAQ VAS (mean, s.d.)	
Overall	46.4 (27.4)
Pain	37.6 (29.9)
Raynaud’s	45.1 (31.3)
Digital ulcers	26.8 (35.0)
Gastrointestinal symptoms	38.6 (35.0)
Respiratory issues	31.3 (31.1)

ARA: anti-RNA polymerase III antibodies; ATA: anti-topoisomerase antibodies; EQ5D5L: EuroQoL 5 dimensions 5 levels; HAQ-DI: HAQ Disability Index; QoL: quality of life; mRSS: Modified Rodnan Skin Score; SSPRO: Scleroderma Skin Patient-Reported Outcome; VAS: visual analogue scale.

### PASTUL scores

Mean PASTUL score at inclusion was 9.2 (s.d. 5.9). PASTUL scores differed between disease subtype, mean score in lcSSc was 7.7 (s.d. 5.5) and 11.5 (s.d. 5.9) in dcSSc (*P* < 0.001). There was no significant correlation between sex, age and disease duration, and PASTUL score.

There was no floor or ceiling effect. The lowest PASTUL score 0 was reported by 7.7% (*n* = 15) of respondents, and 1% (*n* = 2) reported the highest possible score (24).

An overview of all correlations between PASTUL and upper limb mRSS, SSPRO, EuroQol index, Leeds SSc QoL and HAQ-DI scores is provided in Table 2.

**Table 2.** Correlations of PASTUL with upper limb mRSS, EQ5D5L, Leeds SSc HRQoL, HAQ-DI and SSPRO

Outcome measure	Total, *N* = 196		Disease duration <4 years, *N* = 40		dcSSc, *N* = 81	
	ICC	*P*-value	ICC	*P*-value	ICC	*P*-value
Upper limb mRSS	**0.67**	**<0.001**	**0.62**	**<0.001**	**0.69**	**<0.001**
						
Upper limb mRSS at 6 months^a^	**0.78**	**<0.001**	**0.80**	**<0.001**	**0.68**	**<0.001**
*N* = 68						
Upper limb mRSS at 12 months^b^	**0.62**	**<0.001**	**0.80**	**<0.001**	**0.72**	**<0.001**
*N* = 61						
	Pearson’s correlation coefficient		Pearson’s correlation coefficient		Pearson’s correlation coefficient	
EQ5D5L index	**–0.32**	**<0.001**	–0.22	0.254	**–0.40**	**0.003**
EQ5D VAS	–0.20	0.011	**–0.60**	**<0.001**	**–0.25**	**0.040**
Leeds SSc QoL	**0.25**	**<0.001**	**0.53**	**<0.001**	**0.34**	**0.003**
SSPRO total score	0.17	0.027	0.16	0.330	0.23	0.060
SSPRO subdomains						
Physical effects	**0.25**	**<0.001**	0.19	0.238	**0.25**	**0.044**
Physical limitations	0.06	0.420	–0.10	0.950	0.14	0.215
Emotional effects	0.21	0.006	0.30	0.058	0.23	0.068
Social effects	0.09	0.262	–0.02	0.882	**0.28**	**0.022**
HAQ-DI	**0.36**	**<0.001**	**0.56**	**<0.001**	**0.40**	**<0.001**
VAS Overall	**0.40**	**<0.001**	**0.60**	**<0.001**	**0.41**	**<0.001**
VAS Pain	**0.25**	**<0.001**	**0.47**	**<0.001**	**0.33**	**0.003**
VAS Raynaud’s	0.19	0.186	**0.60**	**<0.001**	0.14	0.213
VAS Digital ulcers	**0.34**	**<0.001**	0.22	0.168	**0.30**	**0.007**
VAS GI symptoms	–0.02	0.804	−0.01	0.975	–0.05	0.676
VAS Breathlessness	0.22	0.002	0.111	0.496	0.19	0.101

*P* values <0.05 in bold.

a Compared with PASTUL at 6 months and

b 12 months. PASTUL: Patient self-Assessment of Skin Thickness in Upper Limb; EQ5D5L: EuroQoL 5 dimensions 5 levels; GI: gastrointestinal; HAQ-DI: HAQ Disability Index; QoL: quality of life; ICC: intraclass correlation coefficient; mRSS: modified Rodnan Skin Score; SSPRO: Scleroderma Skin Patient-Reported Outcome; VAS: visual analogue scale.

### Construct validity and reliability

**Figure 1.**
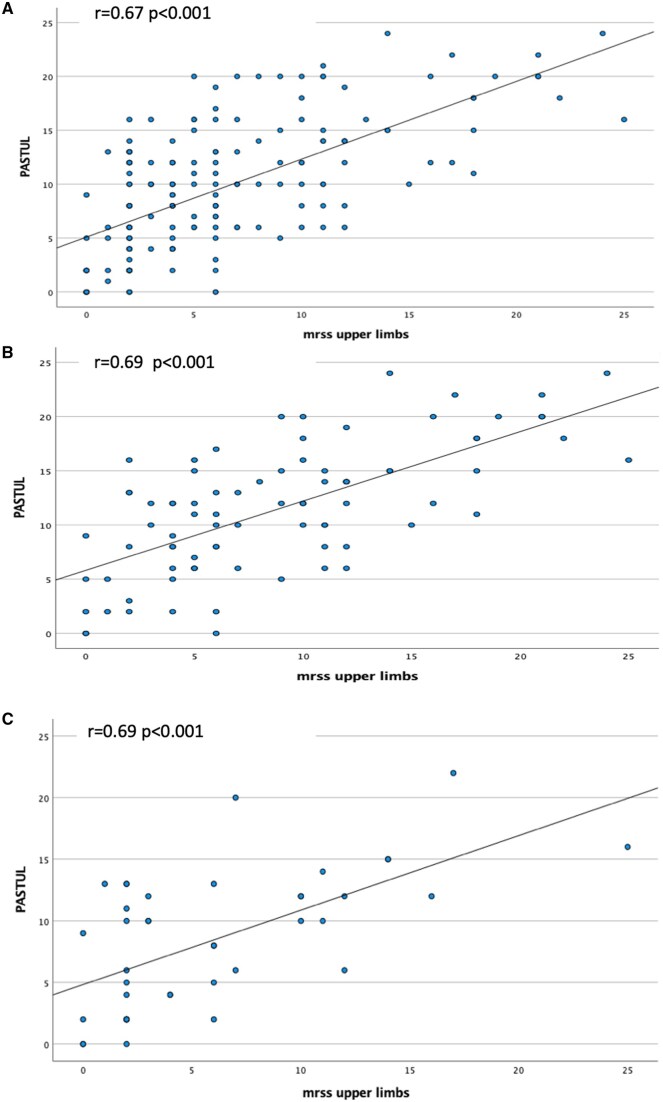
Correlation PASTUL and upper limb mRSS in different groups. PASTUL against upper limb mRSS in (**A**) total group, (**B**) dcSSc patients and (**C**) patients with disease duration of <4 years. PASTUL: Patient self-Assessment of Skin Thickness in Upper Limb; mRSS: modified Rodnan Skin Score

Correlations between PASTUL and mRSS total and upper limbs were strong (r = 0.61 and 0.67, *P* < 0.001, respectively). Correlation between PASTUL and mRSS upper limbs was stronger in dcSSc compared with lcSSc participants (0.69 *vs* 0.51) and in participants <50 years of age (0.77 *vs* 0.58). ICC between mRSS upper limbs and PASTUL is shown in Fig. 1, Table 2 and Supplementary Fig. S3, available at *Rheumatology* online.

ICC between PASTUL and mRSS upper limbs was not different in participants without digital ulcers at time of assessment compared with SSc participants with digital ulcers (r = 0.59 *vs* 0.62).

Test–retest reliability (PASTUL at inclusion and 2 weeks later) was assessed in 93 participants and demonstrated good reliability (ICC of 0.83, *P* < 0.001).

### Feasibility

Feasibility was assessed by 110 participants. Participants scored relevance with a mean score of 3.7 out of 5.0 (s.d. 1.2), clarity of the instructions 4.3 out of 5.0 (s.d. 0.9) and practicability with 4.1 out of 5.0 (s.d. 0.9). The mean time to take the PASTUL was 5.0 min (s.d. 3.6).

Somewhat lower mean scores of practicability were seen in participants >70 years (3.6, s.d. 1.0, respectively) and in participants with digital ulcers (3.7, s.d. 0.9).

CHFS was assessed in a subgroup of participants (*N* = 24). Median CHFS was 13 (IQR 27). Correlation between PASTUL and upper limb mRSS in participants with self-reported impaired hand function (CHFS score >26, *N* = 7) was slightly weaker compared with participants with CHFS <26 (*N* = 17) (ICC = 0.62, *P* = 0.049 and ICC = 0.70, *P* < 0.001, respectively).

### Sensitivity to change

Of the 97 participants included at the Royal Free Hospital London, 68 participants (70%) completed the 6 months visit and 43 participants (44%) the 12 months follow-up visit.

At the follow-up intervals of 6 months 19% of participants (*N* = 17) had a change in mRSS (upper limbs) >3. Change in PASTUL at 6 months interval (baseline to 6 months and 6 months to 12 months follow-up) was not correlated with 6 months change in mRSS (upper limbs) (r = 0.05, *P* = 0.479). At 12 months, 24% of participants (*N* = 10) had a change in mRSS (upper limbs) >3 compared with baseline, which was not correlated with change in PASTUL (r = 0.14, *P* = 0.236). See cross tabs in Table 3.

**Table 3.** Cross tabs change in skin thickening measures with PASTUL or upper limb mRSS at (A) 6-month intervals and (B) 12-month follow-up

(A) Change at 6-month interval between PASTUL and mRSS of upper limbs (*N* = 88)^a^				
		Change mRSS upper limbs >3		
		Decrease	Stable	Increase
Change PASTUL >3	Decrease	22% (2)	13% (9)	13% (1)
	Stable	56% (5)	66% (47)	63% (5)
	Increase	22% (2)	21% (15)	25% (2)

(B) Change at 12 months between PASTUL and mRSS of upper limbs (*N* = 41)^b^				
		mRSS upper limbs change >3		
		Decrease	Stable	Increase
PASTUL change >3	Decrease	0% 0	12% (4)	0 0
	Stable	75% (3)	58% (18)	83% (5)
	Increase	25% (1)	29% (9)	17% (1)

a Cohen’s kappa 0.05, *P* =0.479.

b Cohen’s kappa 0.14, *P* =0.236. PASTUL: Patient self-Assessment of Skin Thickness in Upper Limb; mRSS: modified Rodnan Skin Score.

### Quality of life and other PROs

In our cohort skin thickness measured with PASTUL correlated better with HRQoL outcomes compared with the mRSS (Supplementary Table S4, available at *Rheumatology* online). A stronger correlation was observed between PASTUL and HRQoL in dcSSc, compared with lcSSc, and participants with disease duration <4 years compared with participants with longer disease duration.

There was weak correlation in the total group of participants between PASTUL and mRSS, and EQ5D5L (r = –0.32, *P* = 0.003 and r = –0.20, *P* = 0.023, respectively).

There was a moderate correlation between EQ5D5L index and PASTUL in participants with dcSSc (r = –0.40, *P* = 0.003) and Leeds SSc QoL and PASTUL in participants with early disease (r = 0.53, *P* < 0.001).

HAQ-DI correlated weakly (r = 0.36, *P* < 0.001) with PASTUL in the total group, and moderately in participants with disease duration <4 years (r = 0.56, *P* < 0.001) and dcSSc (r = 0.40, *P* < 0.001). HAQ-DI was strongly correlated with HRQoL (EQ5D5L r = –0.76, *P* < 0.001, Leeds SSc QoL r = 0.70, *P* < 0.001). There was only a weak correlation between HRQoL and skin symptoms reported by the SSPRO (EQ5D5L r = –0.38, *P* < 0.001, Leeds SSc QoL r = 0.35, *P* < 0.001).

## Discussion

In this multicentre study, self-assessment of skin severity in SSc by the PASTUL questionnaire was shown to be reliable, valid and feasible. PASTUL scores showed moderate to strong correlation with the upper limb mRSS and were associated with quality of life in subsets of SSc. This study confirms the results of the pilot study reported by our group during the COVID-19 pandemic [10].

Notably, self-assessed skin thickness was more strongly associated with HAQ-DI and HRQoL in patients with dcSSc and those with early disease [22, 23]. In dcSSc patients, skin thickening is more extensive compared with lcSSc patients and therefore impacts daily life to a larger extent. In early disease, skin thickening may be the predominant symptom and skin score is changing more compared with late disease and could have a larger impact on daily life compared with stable inactive skin later in the disease trajectory [24–26].

Our study demonstrates that high (self-reported) skin scores are associated with lower quality of life and greater limitation in daily functioning, emphasizing the need to address the burden of skin thickening in clinical care as well as the screening and treatment of internal organ involvement.

Interestingly, we observed a better correlation between PASTUL and HRQoL than between mRSS and HRQoL measures. PASTUL is a PRO so it may also partly reflect the impact of skin thickening together with the objective skin thickness, which may align more with other PROs compared with the mRSS performed by a physician.

However, the patient instructions to measure skin thickness separate PASTUL from the QoL measures and although mRSS may be regarded as a more objective measure for physicians, PASTUL may be a more meaningful outcome for patients.

We did not find a correlation between SSPRO and PASTUL, which demonstrates the additional value of a PRO on skin thickening to SSPRO on skin related symptoms.

Interestingly, we observed differences across subgroups with regard to PASTUL and upper limb mRSS, correlations were strong in dcSSc and patients younger than 50 years, but only moderate in lcSSc and above 50 years of age. More extensive skin thickening maybe easier to detect and is more likely to be assessed similarly by patients and physicians compared with limited thickened skin with more subtle skin changes or longstanding skin changes that are not notified as different from normal. The latter was also mentioned by patients in the interviews (Supplementary Data S2, available at *Rheumatology* online). In our study, older age was related to disease subset and disease duration, so this is not an independent determinant of PASTUL performance.

Previous studies have also explored self-assessment of skin in SSc. One Thai study of 23 patients with dcSSc compared the mRSS by patients and physicians and reported moderate correlations at baseline [27]. Participants in this study received mRSS training and had a relatively high mRSS (mean 23.4, s.d. 8.5). In another study of 131 consecutive patients, no training was provided to patients and mean mRSS was 3.6 (s.d. 3.9) [28]. In this study the researchers found a modest correlation between patient and physician assessed skin. Also, no correlation was found between change in mRSS and self-assessment, maybe due to the relatively stable and late-stage patient population, as we observed in our study too.

A strength of PASTUL is the simplified scoring system enabling patients to assess their skin without extensive training and relatively quickly. In our study, most patients, even with digital ulcers, were able to assess their skin adequately. The PASTUL questionnaire could therefore be a useful way to involve patients in the evaluation of disease activity and treatment effects, which could promote patient autonomy and engagement and may also lead to earlier detection of disease progression in between routine hospital visits. In this way, PASTUL fits well in the emerging developments in clinical practice with regard to remote (home) monitoring and telemedicine in rheumatology practice [8, 9, 29].

Importantly, older patients reported lower practicability with PASTUL, which should be taken into account when implementation in clinical practice is considered. The same is the case for patients with impaired hand function, as the, albeit small, subgroup of patients with a higher CHFS showed slightly weaker correlations between PASTUL and upper limb mRSS in our study. Our study has several other strengths and also some limitations. A strength of this study is the prospective design and involvement of four centres, resulting in a large sample size. Secondly, patients received the questionnaire for the skin self-assessment prior to the routine hospital appointment where the mRSS would be done, to prevent influence of the clinician’s assessment on the PASTUL score. In some cases, however, there was a slight delay in completion of the questionnaire, but all clinicians and researchers were instructed to not share the mRSS with the participant while they still needed to complete the PASTUL, ensuring independent assessments. Another strength is that the included patient population is a realistic reflection of a real-world cohort. We were, however, not able to demonstrate sensitivity to change for PASTUL, as the study group was relatively stable. However, as cross-sectional correlations at different time points between PASTUL and upper limb mRSS were well correlated, we believe it will be likely so. This needs to be confirmed in intervention studies, and is currently been done in the ongoing UPSIDE (UPfront autologous hematopoietic Stem cell transplantation versus Immunosuppresive medication in early DiffusE cutaneous systemic sclerosis) trial [30]. The UPSIDE trial is an intervention trial comparing upfront autologous stem cell transplantation with CYC pulse treatment in early dcSSc patients, in which large change in skin thickness after treatment is expected, so sensitivity to change of PASTUL can be further assessed. Furthermore, PASTUL is included in the HANDSOME cohort study (NCT06133244), which follows patients with both early diffuse and limited cutaneous disease, evaluating hand function impairment. In this study impact of hand function impairment on PASTUL performance will be explored.

In conclusion, we showed that the PASTUL questionnaire could be a potential approach for patient to self-assess skin thickness in SSc and provides a useful tool for patient empowerment and home monitoring, and may serve as an outcome measure for clinical trials.

## Supplementary Material

keae561_Supplementary_Data

## Data Availability

Data will be provided by the corresponding author on reasonable request.

## References

[1] Denton CP , Khanna D. Systemic sclerosis. Lancet 2017;390:1685–99. 28413064 10.1016/S0140-6736(17)30933-9

[2] Furst DE , Clements PJ, Steen VD et al The modified rodnan skin score is an accurate reflection of skin biopsy thickness in systemic sclerosis. J Rheumatol 1998;25:84–8. 9458208

[3] Czirják L , Foeldvari I, Müller-Ladner U. Skin involvement in systemic sclerosis. Rheumatology (Oxford) 2008;47(Suppl 5):v44–5. 18784142 10.1093/rheumatology/ken309

[4] Clements PJ , Hurwitz EL, Wong WK et al Skin thickness score as a predictor and correlate of outcome in systemic sclerosis: high-dose versus low-dose penicillamine trial. Arthritis Rheum 2000;43:2445–54. 11083267 10.1002/1529-0131(200011)43:11<2445::AID-ANR11>3.0.CO;2-Q

[5] Herrick AL , Pan X, Peytrignet S et al Treatment outcome in early diffuse cutaneous systemic sclerosis: the European Scleroderma Observational Study (ESOS). Ann Rheum Dis 2017;76:1207–18. 28188239 10.1136/annrheumdis-2016-210503 PMC5530354

[6] Boulos D , Ngian G-S, Rajadurai A et al Long-term efficacy and tolerability of mycophenolate mofetil therapy in diffuse scleroderma skin disease. Int J Rheum Dis 2017;20:481–8. 28337853 10.1111/1756-185X.13035

[7] Namas R , Tashkin DP, Furst DE et al; Participants in the Scleroderma Lung Study I and members of the Scleroderma Lung Study II Research Group. Efficacy of mycophenolate mofetil and oral cyclophosphamide on skin thickness: post hoc analyses from two randomized placebo-controlled trials. Arthritis Care Res 2018;70:439–44. 10.1002/acr.23282 PMC5700860 28544580

[8] Hughes M , Pauling JD, Moore A, Jones J. Impact of Covid-19 on clinical care and lived experience of systemic sclerosis: an international survey from EURORDIS-Rare Diseases Europe. J Scleroderma Relat Disord 2021;6:133–8. 35386739 10.1177/2397198321999927 PMC8892935

[9] Bonfá E , Gossec L, Isenberg DA, Li Z, Raychaudhuri S. How COVID-19 is changing rheumatology clinical practice. Nat Rev Rheumatol 2021;17:11–5. 33139947 10.1038/s41584-020-00527-5 PMC7604913

[10] Spierings J , Ong V, Denton CP. PASTUL questionnaire: a tool for self-assessment of scleroderma skin during the COVID-19 pandemic. Ann Rheum Dis 2021;80:819–20. 33514506 10.1136/annrheumdis-2020-219775

[11] van den Hoogen F , Khanna D, Fransen J et al 2013 classification criteria for systemic sclerosis: an American college of rheumatology/European league against rheumatism collaborative initiative. Ann Rheum Dis 2013;72:1747–55. 24092682 10.1136/annrheumdis-2013-204424

[12] Khanna D , Furst DE, Clements PJ et al Standardization of the modified Rodnan skin score for use in clinical trials of systemic sclerosis. J Scleroderma Relat Disord 2017;2:11–8. 28516167 10.5301/jsrd.5000231 PMC5431585

[13] Steen VD , Medsger TA. The value of the Health Assessment Questionnaire and special patient- generated scales to demonstrate change in systemic sclerosis patients over time. Arthritis Rheum 1997;40:1984–91. 9365087 10.1002/art.1780401110

[14] Reay N. The quality of life in patients with diffuse and limited systemic sclerosis. 2008.

[15] Ndosi M , Alcacer-Pitarch B, Allanore Y et al Common measure of quality of life for people with systemic sclerosis across seven European countries: a cross-sectional study. Ann Rheum Dis 2018;77:1032–8. 29463517 10.1136/annrheumdis-2017-212412 PMC6029637

[16] Feng Y , Devlin N, Herdman M. Assessing the health of the general population in England: how do the three- and five-level versions of EQ-5D compare? Health Qual. Life Outcomes 2015;13:171. 10.1186/s12955-015-0356-8 PMC4618130 26489956

[17] Man A , Correa JK, Ziemek J et al Development and validation of a patient-reported outcome instrument for skin involvement in patients with systemic sclerosis. Ann. Rheum. Dis 2017;76:1374–80. 28213563 10.1136/annrheumdis-2016-210534

[18] Duruöz MT , Poiraudeau S, Fermanian J et al Development and validation of a rheumatoid hand functional disability scale that assesses functional handicap. J. Rheumatol 1996;23:1167–72. 8823687

[19] Daste C , Rannou F, Mouthon L et al Patient acceptable symptom state and minimal clinically important difference for patient-reported outcomes in systemic sclerosis: a secondary analysis of a randomized controlled trial comparing personalized physical therapy to usual care. Semin. Arthritis Rheum 2019;48:694–700. 29685482 10.1016/j.semarthrit.2018.03.013

[20] Portney L , Watkins M. Foundations of clinical research: applications to practice. 2009.

[21] Schober P , Boer C, Schwarte LA. Correlation coefficients: appropriate use and interpretation. Anesth. Analg 2018;126:1763–8. 29481436 10.1213/ANE.0000000000002864

[22] Peytrignet S , Manning J, Wragg E et al Changes in disability and their relationship with skin thickening, in diffuse and limited cutaneous systemic sclerosis: a retrospective cohort study. Scand J Rheumatol 2019;48:230–4. 30394164 10.1080/03009742.2018.1523455

[23] Peytrignet S , Denton CP, Lunt M et al Disability, fatigue, pain and their associates in early diffuse cutaneous systemic sclerosis: the European Scleroderma Observational Study. Rheumatology (Oxford) 2018;57:370–81. 29207002 10.1093/rheumatology/kex410 PMC5850714

[24] LeRoy EC , Black C, Fleischmajer R et al Scleroderma (systemic sclerosis): classification, subsets and pathogenesis. J Rheumatol 1988;15:202–5. 3361530

[25] van Leeuwen NM , Liem SIE, Maurits MP et al Disease progression in systemic sclerosis. Rheumatology (Oxford) 2021;60:1565–7. 33404661 10.1093/rheumatology/keaa911 PMC7937017

[26] Amjadi S , Maranian P, Furst DE et al; Investigators of the D-Penicillamine, Human Recombinant Relaxin, and Oral Bovine Type I Collagen Clinical Trials. Course of the modified Rodnan skin thickness score in systemic sclerosis clinical trials: analysis of three large multicenter, double-blind, randomized controlled trials. Arthritis Rheum 2009;60:2490–8. 19644851 10.1002/art.24681 PMC2725229

[27] Daungkum K , Foocharoen C, Mahakkanukrauh A et al Self-assessment of skin tightness severity by scleroderma patients. Int. J. Rheum. Dis 2016;19:989–95. 27126197 10.1111/1756-185X.12879

[28] Nagy Z , Bálint Z, Farkas H et al Establishment and partial validation of a patient skin self-assessment questionnaire in systemic sclerosis. Rheumatology 2009;48:309–14. 19181657 10.1093/rheumatology/ken490

[29] Avouac J , Marotte H, Balsa A et al Teleconsultation in rheumatology: a literature review and opinion paper. Semin Arthritis Rheum 2023;63:152271. 37813005 10.1016/j.semarthrit.2023.152271

[30] Spierings J , van Rhenen A, Welsing PM et al A randomised, open-label trial to assess the optimal treatment strategy in early diffuse cutaneous systemic sclerosis: the UPSIDE study protocol. BMJ Open 2021;11:e044483. 10.1136/bmjopen-2020-044483 PMC7978271 33737437

