# Supplementary material for: Self-reported skin severity and quality of life in systemic sclerosis: multicentre validation of PASTUL
Source: Rheumatology (Oxford). 2024 Oct 14;64(5):2802–9. doi: 10.1093/rheumatology/keae561 (PMC12048044; doi:10.1093/rheumatology/keae561)
Supplement: keae561_Supplementary_Data [file keae561_supplementary_data.docx]

**Supplementary material**

**S1. Supplementary Figure S1. The PASTUL questionnaire and instruction for patients**

**S2. Supplementary Data S2 (Table S1 and S2). Report on focus groups about PASTUL instructions and implementation**

**S3. Supplementary Figure S3. Validation of PASTUL: Bland-Altman plots**

**S4. Supplementary Table S4. Correlations between HRQoL and skin outcomes.**

**Supplementary Figure S1. PASTUL questionnaire and instructions**

We would like to ask you to assess the skin thickness of the upper limbs, this includes the upper arms, forearms, hands and fingers, again. Please score each individual skin area as follows

**
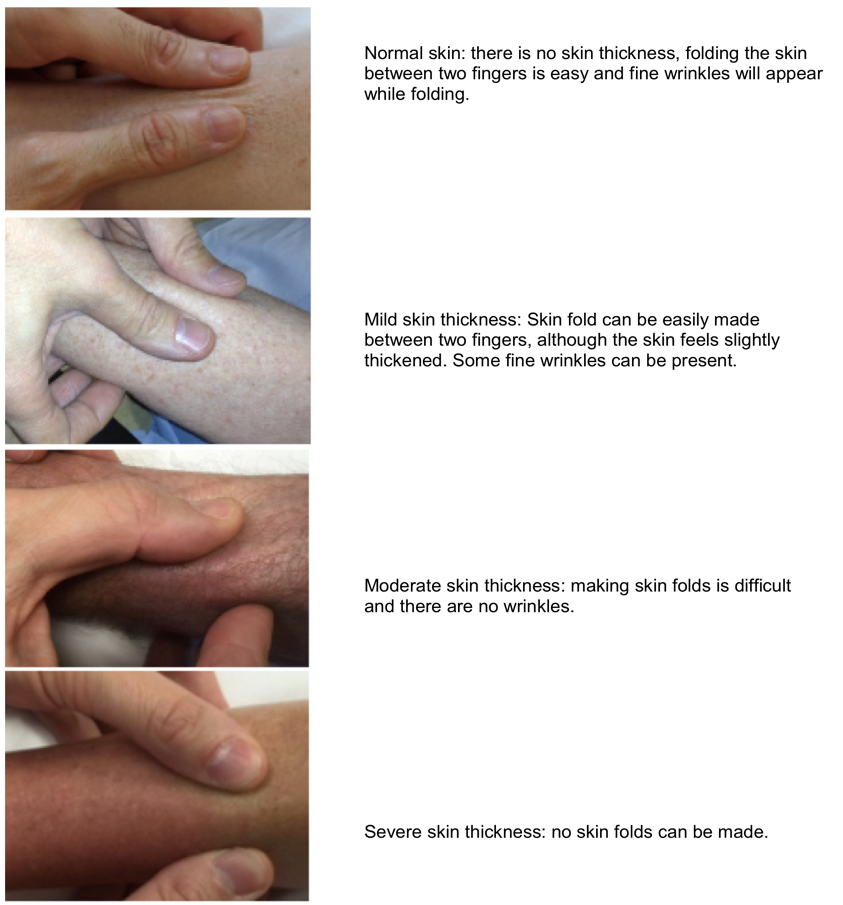
**

| **Area** | **Right** | | | |
| --- | --- | --- | --- | --- |
| Upper arm | Normal | Mild | Moderate | Severe |
| Forearm | Normal | Mild | Moderate | Severe |
| Hand | Normal | Mild | Moderate | Severe |
| Fingers | Normal | Mild | Moderate | Severe |

| **Area** | **Left** | | | |
| --- | --- | --- | --- | --- |
| Upper arm | Normal | Mild | Moderate | Severe |
| Forearm | Normal | Mild | Moderate | Severe |
| Hand | Normal | Mild | Moderate | Severe |
| Fingers | Normal | Mild | Moderate | Severe |

**Supplementary Data S2. Report of focus groups about PASTUL instructions and implementation**

**Design**

Qualitative study with focus groups

**Objective**

To collect feedback about the instruction of the first version of the PASTUL self-assessment questionnaire for systemic sclerosis (SSc) and to explores ways to implement PASTUL in clinical practice.

**Patients**

Patients treated at the Royal Free Hospital, London, United Kingdom, and fulfilling the diagnostic criteria for SSc were eligible for participation. Two rheumatologists invited patients when visiting the hospital for routine check-up or by email, based on professional judgment. Written project information was sent by mail and participation was confirmed over the phone approximately one week later.

**Setting**

In May 2022 three focus groups were conducted online, using MS Teams. The interviews were led by an independent researcher, female, with experience in focus groups (JS). This researcher did not know the participants. The duration of the interviews was 16-34 minutes. All three sessions were audio recorded.

**Data collection**

Background information from participants including sex, age, disease duration, disease subset and presence of digital ulcers was collected. From the partners or relatives, age, sex and the relation to the patient was collected. This information was collected digitally. Participants received an invitation to the online questionnaire (Castor) prior to the focus groups.

One researcher and one rheumatologist developed the interview guide. Participants received an e-mail with instruction about the focus groups and the original version of the PASTUL questionnaire. They were asked to think about the following questions as a preparation for the focus group:
1. Do you have any suggestions for improvement of the instructions for the skin assessment?
2. How would you prefer to take the PASTUL questionnaire?

The interviews were recorded and transcribed verbatim. A report with a summary of the focus groups was send to the participants afterwards.

*Interview guide*

## Instructions for the interviewer - Brief introduction of project - Goals of focus group - Time schedule meeting - Rules and instructions for voting

## Questions

1. Do you have any comments on the instructions of the PASTUL questionnaire?
2. Do you have any comments on the layout of the PASTUL questionnaire?
3. In which way would you prefer to receive the PASTUL questionnaire?

   *Instructions for the interviewer: give suggestions / examples: paper document, e-mail with link to website, mobile application.*
4. Do you currently use any other health-related applications?

a. If yes, what is your experience with these applications?

b. If no, why don’t you use any applications?

1. In case the PASTUL questionnaire would be available on a mobile application, would you download this app and use the app?
2. Would you prefer the PASTUL integrated in an existing application or in a separate app?

Closing

1. Do you have any additional comments?
2. Would you be willing to evaluate the PASTUL questionnaire in a mobile application?

*Instructions for the interviewer: Interviewer will briefly summarize the results of the meeting and will explain that all participants will receive a report from the focus group session. The questionnaire will be adjusted accordingly and shared with the panel for final agreement.*

**Data analysis**

The collected data will be assessed with descriptive analysis.

**Ethics**

This study was approved by the REC or Royal Free Hospital. All participants provided written consent.

**Results**

*Patients*

For the focus interviews, ten patients were included. There were groups with three participants and one with four participants. In total nine patients (90%) and one partner (10%) participated. Characteristics of the participants are shown in supplementary tables S1.

**Supplementary Table S2: Patient characteristics focus groups**

|  | **Participants (N=10)** |
| --- | --- |
| Age, years; median (IQR) | 45.0 (34) |
| Male, % (N) | 50% (5) |
| Disease duration, years; median (IQR) | 4.5 (2) |
| Disease subset  LcSSc DcSSc Unknown | 20% (2) 50% (5) 20% (2) |
| Digital ulcers; % (N) | 20% (2) |
| PASTUL score; median (IQR) | 15 (11) |
| VAS relevance PASTUL (1-5); median (IQR) | 3 (3) |
| VAS understandable PASTUL (1-5); median (IQR) | 4 (3) |
| VAS feasibility PASTUL (1-5); median (IQR) | 4 (3) |
| Time needed for PASTUL, minutes; median (IQR) | 5 (6) |

Abbreviations: dcSSc: diffuse cutaneous systemic sclerosis, IQR: interquartile range, lcssc; limited cutaneous systemic sclerosis, PASTUL: patient self-assessment of scleroderma skin thickness in upper limb, VAS: visual analog scale

*Skin self-assessment*

All participants thought the instructions of the PASTUL questionnaire including the pictures with related scores were clear enough to do an assessment of skin thickness. The pictures were found to be helpful.

They also thought this questionnaire gave insight in what their rheumatologists were doing when assessing their skin during study visits or routine care visits.

Presence of carpal tunnel syndrome, digital ulcers and fake nails were believed to limit self-assessment.

Participants reported it would be helpful to have another person assess their skin, as they felt they did not know normal skin anymore so self-assessment could be less accurate. They did not feel uncomfortable to let another person close to them (i.e. partner or family member) assess their skin.

*Implementation*

All participants preferred to do the PASTUL online on a website on their computer or laptop. This was preferred over paper questionnaires. They thought receiving an invitation by email every now and then would be best.

Most participants did not prefer to use a mobile application. Reasons that were given for this was the difficulty to use a smart phone because of the small screen and touch screen. Also, some participants thought it was complicated to download and use applications on their phone. One patient mentioned he forgets to use mobile applications despite receiving notifications about it.

Three participants had other mobile applications on their phone, all three were using an application to make appointments with their general practitioner.

Four participants mentioned they would try the application in case the PASTUL questionnaire would only be available on a mobile application. They were more likely to use the application if it was combined with information about SSc or Raynaud’s phenomenon or other health features.

**Supplementary Table S3: Representative quotes**

| **Skin self-assessment and PASTUL** | **Implementation** |
| --- | --- |
| *“I am quiet happy to do it myself, I haven’t been at the clinics for a long time, so it gives me some idea on how things are going.”* | “*I prefer to use a keyboard and large screen for questionnaires, it much more convenient than a mobile phone.”* |
| *“Instructions were all fine, pictures made it very clear.”* | *“Using e-mail and computer is easier.”* |
| *“It was a bit hard to do the score because of a digital ulcer on my thumb and my fake nails which are very long.”* | *“Many people have different preferences, it would be great if there would be both an online invitation for computer and an application.”* |
| *“It would be really helpful to have another person assessing the skin. Good to have someone close to you helping you, because you do not know what is normal anymore, you get used to your skin being different. That is why the images are also helpful.”* | *“I use Patient Access/NHS, an application from the GP office for prescriptions and appointments. If scleroderma related stuff can be combined it would be more attractive to use.”* |
| *“We get used to our bodies and skin, we do not notice thickening or changes anymore, so assessment by someone close to you will be valuable.”* | *“I have a few health applications on my phone but I don’t use them. Maybe my kids could help me with it if necessary.”* |
| *“My partner would probably do a better job assessing my skin.”* | *“Every application is slightly different, so you have to get used to it.”* |

**S3. Supplementary Figure S3. Validation of PASTUL: Bland-Altman plots**Bland-Altman plots of difference between mRSS upper limbs and PASTUL in total group (A), dcSSc participants (B) and participants with disease duration of < 4 years (C).

1. **
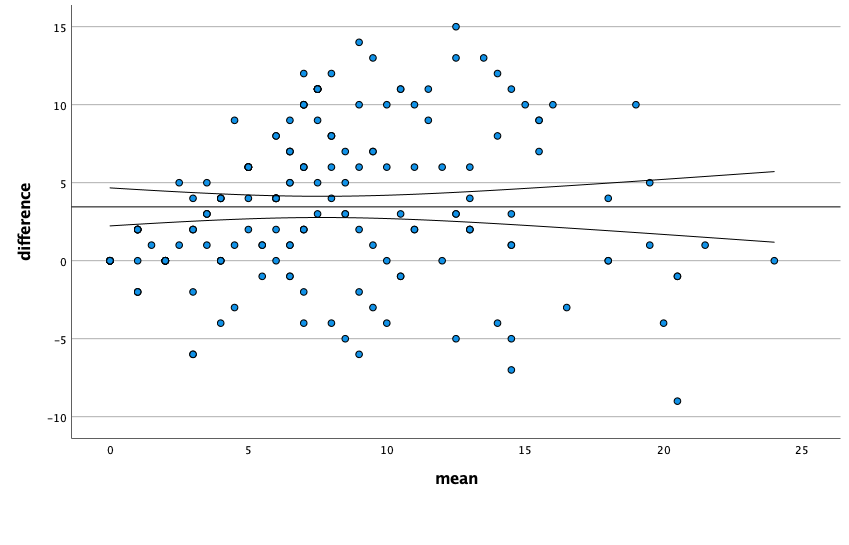
**

**
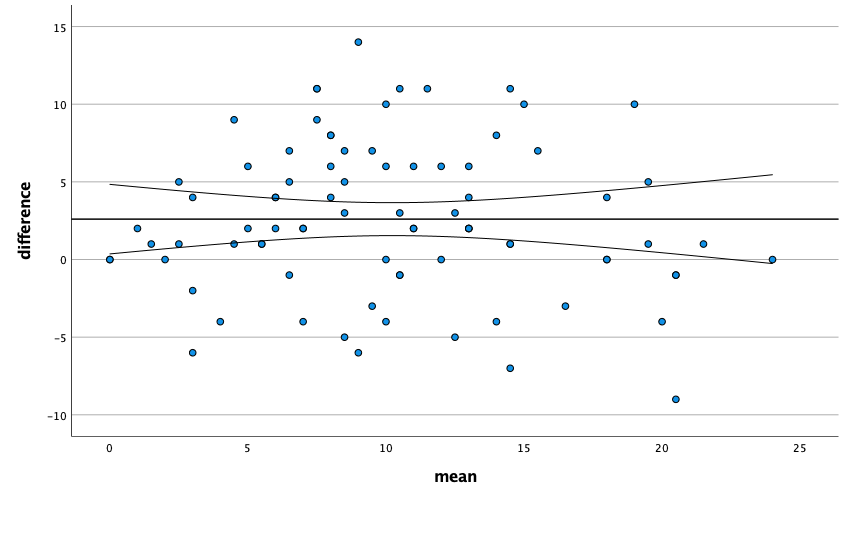
B.**


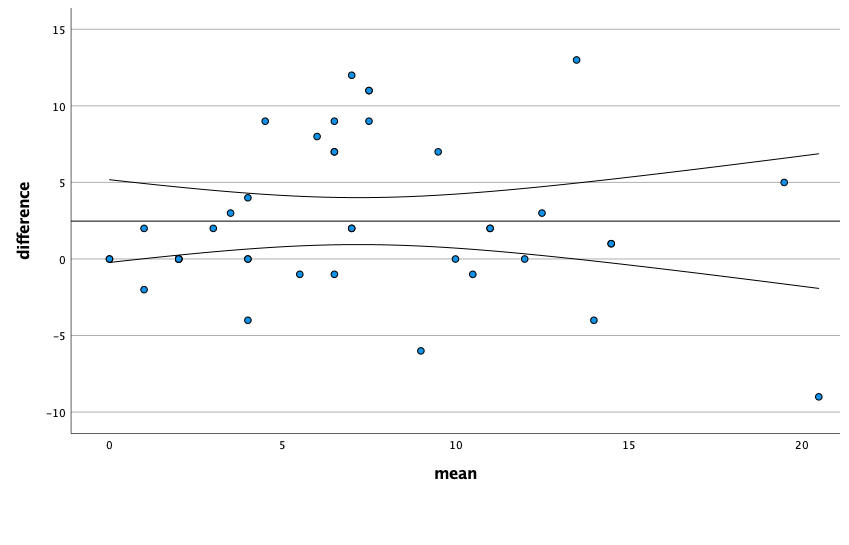
**C.**

**S4. Supplementary Table S4. Correlations between HRQoL and skin outcomes.

a. Correlation between EQ5D5L index and skin outcome measures**

| **Outcome measure** | **Total**  **N=196** | | **Disease duration  < 4 years, N=40** | | **Diffuse cutaneous SSc**  **N=81** | |
| --- | --- | --- | --- | --- | --- | --- |
|  | Pearson’s correlation coefficient | P value | Pearson’s correlation coefficient | P value | Pearson’s correlation coefficient | P value |
| **PASTUL** | -0.32 | **0.003** | -0.22 | 0.254 | -0.40 | **0.003** |
| **mRSS baseline** | -0.20 | **0.023** | -0.08 | 0.699 | -0.08 | 0.527 |
| **mRSS upper limbs** | -0.22 | **0.012** | -0.03 | 0.880 | -0.09 | 0.527 |
| **SSPRO total score** | -0.38 | **<0.001** | -0.28 | 0.245 | -0.18 | 0.359 |
| **SSPRO subdomains** |  |  |  |  |  |  |
| **Physical effects** | -0.41 | **<0.001** | -0.33 | 0.312 | -0.24 | 0.105 |
| **Physical limitations** | -0.22 | **0.014** | -0.23 | 0.130 | -0.06 | 0.702 |
| **Emotional effects** | -0.37 | **<0.001** | -0.22 | 0.278 | -0.17 | 0.241 |
| **Social effects** | -0.35 | **<0.001** | -0.23 | 0.234 | -0.21 | 0.338 |
|  |  |  |  |  |  |  |
| **HAQ DI** | -0.76 | **<0.001** | -0.73 | **<0.001** | -0.73 | **<0.001** |
| **VAS Overall** | -0.66 | **<0.001** | -0.69 | **<0.001** | -0.59 | **<0.001** |
| **VAS Pain** | -0.67 | **<0.001** | -0.68 | **<0.001** | -0.53 | **<0.001** |
| **VAS Raynaud’s** | -0.50 | **<0.001** | -0.44 | **0.019** | -0.41 | **0.003** |
| **VAS Digital ulcers** | -0.44 | **<0.001** | -0.29 | 0.134 | -0.39 | **0.005** |
| **VAS GI symptoms** | -0.51 | **<0.001** | -0.57 | **0.001** | -0.42 | **0.002** |
| **VAS Breathlessness** | -0.56 | **<0.001** | -0.33 | 0.090 | -0.57 | **<0.001** |

**b. Correlation between Q5D VAS index and skin outcome measures**

| **Outcome measure** | **Total**  **N=196** | | **Disease duration < 4 years, N=40** | | **Diffuse cutaneous SSc**  **N=81** | |
| --- | --- | --- | --- | --- | --- | --- |
|  | Pearson’s correlation coefficient | P value | Pearson’s correlation coefficient | P value | Pearson’s correlation coefficient | P value |
| **PASTUL** | -0.20 | **0.011** | -0.60 | **<0.001** | -0.40 | **0.003** |
| **mRSS baseline** | -0.13 | 0.092 | -0.38 | **0.017** | -0.11 | 0.364 |
| **mRSS upper limbs** | -0.16 | **0.049** | -0.38 | **0.017** | -0.13 | 0.290 |
| **SSPRO total score** | -0.19 | 0.181 | -0.28 | 0.245 | -0.14 | 0.275 |
| **SSPRO subdomains** |  |  |  |  |  |  |
| **Physical effects** | -0.18 | 0.200 | -0.10 | 0.529 | -0.18 | 0.166 |
| **Physical limitations** | -0.08 | 0.072 | -0.00 | 0.979 | -0.00 | 0.991 |
| **Emotional effects** | -0.21 | **0.008** | -0.13 | 0.420 | -0.13 | 0.241 |
| **Social effects** | -0.23 | **0.004** | -0.23 | 0.234 | -0.26 | **0.044** |
|  |  |  |  |  |  |  |
| **HAQ DI** | -0.31 | **<0.001** | -0.67 | **<0.001** | -0.26 | **0.038** |
| **VAS Overall** | -0.34 | **<0.001** | -0.65 | **<0.001** | -0.22 | **0.073** |
| **VAS Pain** | -0.40 | **<0.001** | -0.55 | **<0.001** | -0.32 | **0.009** |
| **VAS Raynaud’s** | -0.41 | **<0.001** | -0.50 | **0.001** | -0.41 | **<0.001** |
| **VAS Digital ulcers** | -0.20 | **0.010** | -0.38 | **0.019** | -0.01 | 0.946 |
| **VAS GI symptoms** | -0.18 | **0.026** | -0.26 | 0.120 | -0.01 | 0.924 |
| **VAS Breathlessness** | -0.23 | **0.004** | -0.40 | **0.013** | -0.12 | **0.360** |

**c. Correlation between Leeds SSc HRQoL index and skin outcome measures**

| **Outcome measure** | **Total**  **N=196** | | **Disease duration < 4 years N=40** | | **Diffuse cutaneous SSc**  **N=81** | |
| --- | --- | --- | --- | --- | --- | --- |
|  | Pearson’s correlation coefficient | P value | Pearson’s correlation coefficient | P value | Pearson’s correlation coefficient | P value |
| **PASTUL** | 0.25 | **<0.001** | 0.53 | **<0.001** | 0.34 | **0.003** |
| **mRSS baseline** | 0.30 | **<0.001** | 0.30 | 0.060 | 0.34 | **0.003** |
| **mRSS upper limbs** | 0.28 | **<0.001** | 0.28 | 0.076 | 0.29 | **0.009** |
| **SSPRO total score** | 0.35 | **<0.001** | 0.27 | 0.096 | 0.34 | **0.006** |
| **SSPRO subdomains** |  |  |  |  |  |  |
| **Physical effects** | 0.38 | **<0.001** | 0.32 | **0.046** | 0.37 | **0.003** |
| **Physical limitations** | 0.27 | **<0.001** | 0.19 | 0.243 | 0.28 | **0.024** |
| **Emotional effects** | 0.35 | **<0.001** | 0.28 | 0.077 | 0.30 | **0.015** |
| **Social effects** | 0.31 | **<0.001** | 0.122 | 0.454 | 0.31 | **0.012** |
|  |  |  |  |  |  |  |
| **HAQ DI** | 0.70 | **<0.001** | 0.74 | **<0.001** | 0.68 | **<0.001** |
| **VAS Overall** | 0.74 | **<0.001** | 0.65 | **<0.001** | 0.75 | **<0.001** |
| **VAS Pain** | 0.68 | **<0.001** | 0.85 | **<0.001** | 0.60 | **<0.001** |
| **VAS Raynaud’s** | 0.58 | **<0.001** | 0.64 | **<0.001** | 0.42 | **<0.001** |
| **VAS Digital ulcers** | 0.38 | **<0.001** | 0.31 | 0.054 | 0.35 | **0.002** |
| **VAS GI symptoms** | 0.44 | **<0.001** | 0.43 | **0.006** | 0.31 | **0.006** |
| **VAS Breathlessness** | 0.42 | **<0.001** | 0.45 | **0.004** | 0.46 | **<0.001** |

*Footnotes: P values <0.05 in bold.*

Abbreviations: dcSSc: diffuse cutaneous systemic sclerosis, EQ5D5L: EuroQoL 5 dimensions 5 levels, GI: gastrointestinal, HAQ DI: health assessment questionnaire disability index, HRQoL: health related quality of life, mRSS: Modified Rodnan Skin Score, SSc: systemic sclerosis, SSPRO: scleroderma skin patient reported outcome, VAS: visual analog scale.
